# “Taking Charge” after a diagnosis of cognitive impairment or dementia: a randomized controlled trial

**DOI:** 10.1093/geront/gnaf280

**Published:** 2025-11-24

**Authors:** Miia Rahja, Harry McNaughton, Maria Crotty, Aarti Gulyani, Qunyan Xu, Owen Davies, Elita Santosaputri, Kate Laver

**Affiliations:** Flinders University, College of Medicine and Public Health, Flinders Health and Medical Research Institute, Adelaide, South Australia, Australia; Medical Research Institute of New Zealand, Wellington, New Zealand; Flinders University, College of Medicine and Public Health, Flinders Health and Medical Research Institute, Adelaide, South Australia, Australia; Division of Rehabilitation, Aged and Palliative Care, Southern Adelaide Local Health Network, Adelaide, South Australia, Australia; Flinders University, College of Nursing and Health Sciences, Caring Futures Institute, Adelaide, South Australia, Australia; Flinders University, College of Nursing and Health Sciences, Caring Futures Institute, Adelaide, South Australia, Australia; Division of Rehabilitation, Aged and Palliative Care, Southern Adelaide Local Health Network, Adelaide, South Australia, Australia; Division of Rehabilitation, Aged and Palliative Care, Southern Adelaide Local Health Network, Adelaide, South Australia, Australia; Division of Rehabilitation, Aged and Palliative Care, Southern Adelaide Local Health Network, Adelaide, South Australia, Australia; Flinders University, College of Nursing and Health Sciences, Caring Futures Institute, Adelaide, South Australia, Australia

**Keywords:** Take Charge, Randomized controlled trial, Self-management, Mild cognitive impairment, Dementia

## Abstract

**Background and Objectives:**

Self-directed programs can steer positive trajectories after life-changing health events. This study evaluated if one such program (Take Charge) could improve quality of life (QoL) after diagnosis of mild cognitive impairment (MCI) or mild dementia compared with lifestyle education.

**Research Design and Methods:**

Two-arm, open-label, randomized controlled trial including people with MCI or mild dementia in Adelaide, Australia. Participants were assigned to Take Charge intervention (*n* = 80) or control group (lifestyle education with waitlist option) (*n* = 80). The primary outcome was QoL measured using the Physical Component Summary (PCS) score of the Short Form 36 (SF-36) at three months following baseline comparing Take Charge to control. Secondary outcomes included the mental component summary (MCS) score of SF-36, hope, mood (depression scale), and activity engagement at three and six months. A subset of participants participated in an interview about their experiences with Take Charge.

**Results:**

Participants had a mean age of 79.6 years (*SD* 7.2) and 51.2% were female. The mean Mini-Mental State Examination score was 24.2 (*SD* 3.2). No significant differences were found in PCS scores between groups at three months (−0.53; 95% CI: −4.23, 3.17; *p* = .78). However, interviews suggested that those actively engaged in Take Charge felt more positive about the future.

**Discussion and Implications:**

There was no significant difference between the Take Charge and control groups, and Take Charge did not improve physical health-related QoL for people living with MCI or mild dementia. More work is needed to establish the appropriateness and efficacy of self-directed programs for this population.

## Background

Approximately 244 people are diagnosed with dementia in Australia daily (Brown et al., 2017). Most are diagnosed when they have mild symptoms meaning they can still carry out basic everyday activities and social functions (Knopman & Petersen, 2014). Mild cognitive impairment (MCI) which involves cognitive decline beyond normal aging, can precede dementia and affects 12%–18% of people aged ≥60 years (Alzheimer’s Association, 2022; Winblad et al., 2004). Approximately 10% of people diagnosed with MCI progress to dementia within a year (Chen et al., 2017).

A key challenge after an MCI or dementia diagnosis is coping with the prospect of multiple losses, including self-esteem, function, and social roles (Robinson et al., 2011). Current care often focuses on adaptation and compensation which can disempower individuals (Low et al., 2018). Research from the United Kingdom shows that people living with dementia seek to maintain hope and improve their quality of life (QoL) after diagnosis (Wolverson et al., 2010). In 2019, Dementia Australia conducted focus groups with 137 people living with dementia and carers, identifying the need for post-diagnostic supports that foster hope, engagement and participation in meaningful activities (Dementia Australia, 2019).

In Australia, current practice does not sufficiently support people after receiving a diagnosis of MCI or dementia (Royal Commission into Aged Care Quality and Safety, 2021). Some Government funded post-diagnostic support is available, but these supports focus primarily on education, counselling and advisory services and they are primarily offered via the telephone. A case management approach may be offered, and while it can deliver some benefit, it is resource intensive, and continuity of care is difficult to maintain due to high staff turnover (Reilly et al., 2015; Royal Commission into Aged Care Quality and Safety, 2021). Case management also typically focuses on education, compensation, and adaptation rather than promoting hope, independence, and QoL. Dementia consumer groups have called for services which seek to improve QoL to fill the gap post-diagnosis (Dementia Australia, 2019).

Take Charge is a short (two-session) therapeutic intervention that encourages people with chronic health conditions to consider their values, what is important to them, and how to maximize their QoL in the coming months (Fu et al., 2020). Take Charge was developed based on existing evidence and underpinning theory related to self-determination and intrinsic motivation (McNaughton et al., 2023; Ryan et al., 1997). Thus, it considers people’s inherent growth tendencies and innate psychological needs that are the basis for their self-motivation and personality integration, as well as the conditions that foster those positive processes (Ryan et al., 1997). The intervention has been tested with stroke survivors in two randomized controlled trials in New Zealand (Fu et al., 2020; Harwood et al., 2012). In the largest trial (*n* = 400) 38% of participants had cognitive problems and 49% had a communication disorder but there was a similar response to the intervention with or without cognitive or communication issues. Both trials showed that people who received the intervention reported better health-related QoL and were less likely to be physically dependent 12 months following the intervention (Fu et al., 2020; Harwood et al., 2012). Evaluations of the Take Charge intervention with other populations are currently underway with a pilot study conducted in people with chronic obstructive pulmonary disease (Levack et al., 2023) and a randomized trial with people with Long COVID in progress (Luker et al., 2025). Interventions such as Take Charge may play a critical role in steering positive trajectories and is consistent with the types of programs identified to be of value by people with dementia (Dementia Australia, 2019).

The purpose of this study was to test the effects of Take Charge for people recently diagnosed with MCI or mild dementia in a primary care or memory clinic setting. The researchers hypothesized that people who participated in Take Charge would report higher levels of QoL, higher levels of hope, better mood, and increased levels of independence three months after participating in the intervention compared with those who were placed on a wait-list and received lifestyle education.

## Research design and methods

This open label randomized controlled trial compared two active forms of treatment. The intervention group received Take Charge and the control group received dose-matched lifestyle education and was given the option to be placed on a waitlist for Take Charge. A study protocol was developed *a priori*, and this study was registered with the Australian New Zealand Clinical Trials Registry (ID: ACTRN12621000282886). Ethical approval was obtained from the Southern Adelaide Local Health Network Human Research Ethics Committed (ID: 2021HRE0009). All participants provided written informed consent to participate.

### Consumer involvement

This study was guided by Dementia Australia’s “Our Solution: Quality Care for People Living with Dementia,” developed through extensive consultation with people living with dementia and their families. Consumer representative (JJ), a former carer of someone with younger-onset dementia, was involved throughout to integrate the consumer perspective from planning to reporting.

### Setting

The study was conducted in Adelaide, South Australia. Participants were recruited from older person and memory clinics in a tertiary teaching hospital, a rehabilitation hospital, private practitioner referrals, and through a community aged care provider.

### Participants

Adults (≥18 years) diagnosed within the past six months with mild cognitive impairment (MCI, MMSE >23) or mild dementia (MMSE 19–23) by a geriatrician or physician were eligible. Non-English speakers could participate with family or ­interpreter support. Those with life-limiting conditions and <12 months’ life expectancy were excluded. As the intervention is person-centered and focuses on the individual’s values and priorities, the presence of, or involvement of a care partner was optional and based on the preference of the person with MCI or dementia. If a care partner (≥18 years) was closely involved in providing care to the participant, they were invited to participate with the participant and asked to contribute to a survey regarding their care provision.

Based on Fu et al.’s (2020), Take Charge trial in stroke survivors, we powered the study using the SF-36 Physical Component Summary (PCS) score (Hays et al., 1993). Given an undefined minimal clinically important difference in stroke, Fu et al. used a five-point threshold. To reflect smaller but meaningful changes in MCI/dementia, we set a four-point difference (mean 47.3, *SD* 8.4). With α = 0.05 and power = 0.8, we required 138 participants, adjusting to 160 for ∼10% dropout.

### Interventions and study procedures

Health professionals at participating sites identified potential participants, provided study flyers, and, with consent, shared contact details with researchers. A research team member contacted potential participants to discuss the study and provided a participant information sheet. After obtaining consent, participants completed baseline assessments and were randomized to either the Take Charge or the control group.

#### Take Charge intervention

The Take Charge intervention involved two consultations (up to 60 min each) with a health professional, held six weeks apart, in participants’ homes or their preferred locations. Family members could attend upon request. Sessions focused on self-reflection, goal setting, and action planning, supported by an illustrated workbook. More details on the original Take Charge intervention and materials are available at www.mrinz.ac.nz/programmes/stroke. Adaptations from the stroke version replaced “My Stroke” with “My Diagnosis” and modified sections on understanding the condition. Participants kept the workbook.

Two trained health professionals (nursing/social work backgrounds) with experience in aged care delivered the intervention. Training included a one-day workshop, a manual, and ongoing clinician support.

#### Control intervention

The control group received two equivalent 60-min sessions, six weeks apart, focusing on lifestyle education with written materials from Dementia Australia (https://www.dementia.org.au/brain-health/reducing-your-risk-dementia). Topics included brain and heart health, nutrition, and social connections. The same staff delivered both interventions. Control participants were offered Take Charge after completing outcome assessments.

### Randomization and masking

Participants were randomized into the two study groups in a 1:1 ratio. The randomization schedule was developed using a computer-generated program by an independent researcher and this information was transferred to sequentially numbered, opaque, sealed envelopes. Study participants were stratified by diagnosis: dementia and MCI. While study participants or the person providing the intervention could not be blinded, the outcome assessors were blinded.

### Outcomes of interest

Participants completed four outcome measures (1) Short form 36 (SF-36) (Hays et al., 1993), (2) Frenchay Activities Index (FAI) (Schuling et al., 1993), (3) Adult Hope Scale (AHS) (Snyder et al., 1991), and (4) the 15-item Geriatric Depression Scale (GDS-15) (Sheikh & Yesavage, 1986), with a description and scoring methods in Supplementary Table S1. The primary outcome was health-related QoL, measured by the SF-36 PCS score at three months post-baseline comparing the Take Charge intervention with control group. SF-36 is a widely used, self-reported, QoL measure for assessing care outcomes in adults. The PCS was selected due to its relevance to the intervention’s goal of enhancing QoL, including dimensions like general health and physical capability. Secondary outcomes included SF-36 MCS, FAI, AHS, and GDS scores at three and six months, chosen to complement the PCS.

At three months, participants rated satisfaction via six Likert-scale questions, with additional feedback provided (Supplementary Table S1). At six months, health service use, and time spent caregiving were assessed with a revised Resource Utilization in Dementia (RUD-Lite) (Wimo & Winblad, 2003) questionnaire. Completion of the RUD-Lite instrument requires good memory (recalling episodes of health care use over a three month period), thus care partners (with appropriate consent) were used as informants for this questionnaire if a participant was agreeable for their involvement.

All baseline assessments were face-to-face, and three- and six-month outcomes were assessed by trained research assistants via phone. If participants were unreachable, surveys were mailed or emailed. A subgroup of participants was invited to a face-to-face interview about their intervention experience. The researchers aimed to interview participants until data saturation was reached. The interview guide is in the Supplementary Material.

### Data analysis

Detailed steps for scoring each outcome are provided in Supplementary Table S1. The primary outcome, the standardized SF-36 PCS score, was calculated using Australian general population norms (Australian Bereau of Statistics, 1995). Subscale scores for PCS and MCS were averaged if ≥50% of items had valid responses (Kosinski et al., 2000). A similar approach was applied to handle missing data for secondary outcomes (FAI, AHS, and GDS). Less than 2% of data were missing at random at baseline (Supplementary Table S2), and no imputation was used due to some missing data being not at random.

The baseline characteristics were expressed as mean (standard deviation, *SD*) for continuous data and frequencies (percentages) for categorical data, with statistical tests comparing groups by diagnosis (dementia vs MCI). Summary statistics for outcomes were calculated at baseline, three, and six months. A linear mixed-effects model assessed between-group (fixed) and within-group (random) effects of the Take Charge intervention versus control at different time points. This method was used due to repeated measurements of participants’ outcomes at different time points as it measured the variability within participants and intra-class correlation coefficient (ICC). Two models were used for the analysis. Model 1 included treatment, time, and interactions; the adjusted Model 2 added covariates: diagnosis (dementia/MCI), age, gender, living situation, and cognitive impairment (MMSE ≤23/MMSE >23).

Subgroup analyses were planned for outcomes at three months by diagnosis and baseline characteristics (age, gender, living situation, cognitive impairment) for the primary outcome. Interaction tests were conducted to assess if covariates influenced outcomes over time, with significance adjusted using Bonferroni correction. Care partner data and health service utilization were compared between groups at six months. Data analysis was performed using STATA Version 18.0.

The interviews were analyzed using an inductive thematic approach (Braun & Clarke, 2006). Transcripts were read and re-read to generate codes for key topics, which were then grouped into high-level themes. These themes were refined to ensure they captured the key messages related to the research questions. Representative quotes were selected to illustrate each theme. The primary analysis was conducted by Miia Rahja, with Marina Brown and Kate Laver reviewing the transcripts and noting initial codes. High agreement was found between coders. Regular meetings among Miia Rahja, Kate Laver, Marina Brown, and Maria Crotty facilitated discussion of emerging data, resolving coding disagreements and ensuring diverse perspectives.

## Results

**Figure 1.**
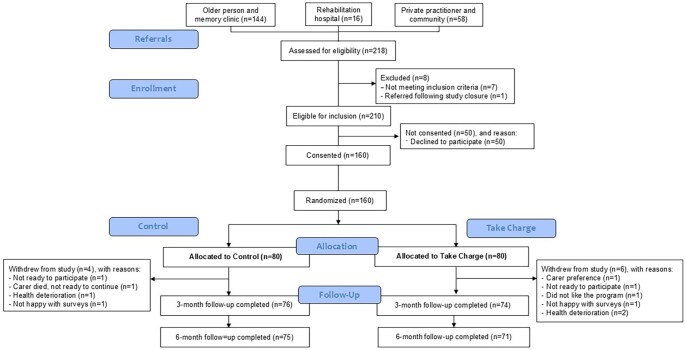
Consort diagram.

Participants were recruited between June 28, 2021 and May 23, 2023. A CONSORT diagram of the participant recruitment is provided in Figure 1. A total of 218 referrals were received, of which 210 (96.3%) were eligible for inclusion. Of the 210 potential participants approached for the study, 160 (76.2%) agreed to take part. Of the 160 participants, 10 withdrew from the study and four could not be reached for their six-month assessment.

Participant and care partner characteristics for the entire cohort and for the intervention and control groups are depicted in Table 1. The mean age was 79.6 years (*SD* 7.2 years, range 52–96 years), over half (*n* = 93, 58.1%) of the participants had dementia, and were women (*n* = 82, 51.2%). The intervention and control groups were balanced and no significant differences between the groups were found at baseline. Participant and care partner characteristics for the entire cohort, individuals with dementia and MCI (stratification factor) are depicted in Supplementary Table S3. Thirteen (8.1%) participants identified as coming from Culturally and Linguistically Diverse or Aboriginal and Torres Strait Islander backgrounds. No adverse events were reported.

**Table 1.** Baseline participant and carer characteristics by trial treatment.

Characteristics	Overall	Take charge	Control	*p*-value
**Number of participants in each treatment group, ** *n*	160	80	80	
**Participant related**				
**Age of participant (years), mean (*SD*)**	79.6 (7.2)	79.0 (7.6)	80.1 (6.8)	.36
**Gender of participant, *n* (%)**				.75
** Female**	82 (51.2%)	42 (52%)	40 (50%)	
** Male**	78 (48.8%)	38 (48%)	40 (50%)	
**Mimi-mental state examination (MMSE) score, mean (*SD*)**	24.2 (3.2)	24.0 (3.3)	24.3 (3.0)	.65
**Living situation, *n* (%)**				.85
** Alone**	39 (24.4%)	20 (25%)	19 (24%)	
** With family**	121 (75.6%)	60 (75%)	61 (76%)	
**Diagnosis, *n* (%)**				.87
** Dementia**	93 (58%)	47 (59%)	46 (57%)	
** MCI**	67 (42%)	33 (41%)	34 (42%)	
**Carer partner related**				
**Age of care partner (years), mean (*SD*)**	70.6 (11.8)	70.2 (12.1)	70.9 (11.6)	.70
**Gender of care partner, *n* (%)**				.80
** Female**	90 (65.7%)	42 (65%)	48 (67%)	
** Male**	47 (34.3%)	23 (35%)	24 (33%)	
**Relationship to patient, *n* (%)**				.15
** Spouse/partner**	92 (67.2%)	43 (66%)	49 (68%)	
** Sibling**	1 (0.7%)	0 (0%)	1 (1%)	
** Children**	39 (28.5%)	17 (26%)	22 (31%)	
** Friends**	1 (0.7%)	1 (2%)	0 (0%)	
** Other**	4 (2.9%)	4 (6%)	0 (0%)	

Abbreviations. MCI, mild cognitive impairment; MMSE, mini mental status examination.

Note. *p*-Values obtained using chi-squared test for categorical variables or two-sample *t*-test for continuous variables.

Table 2 shows the mean baseline and three-month scores for the primary and secondary outcomes, along with adjusted mean differences (MD) at three months, calculated using a linear mixed model. Summary statistics on subscales for all outcome measures are reported in Supplementary Table S4 and Table S5 presents the MD at six months (for Models 1 and 2). The MD in physical health-related QoL (SF-36 PCS score) at three months was 0.53 points lower in the Take Charge group compared to the control group (95% CI: −4.23 to 3.17), but this difference was not significant (*p* = .78; Table 2). We also found no significant differences in the mean PCS scores after adjusting for treatment and time interactions, diagnosis and baseline characteristics (Model 2, MD: −0.78 [95% CI: −4.48 to 2.91], *p* = .68). Similarly, we found no significant differences in the PCS scores at six months (with or without adjustments, Supplementary Table S5). The adjusted MCS scores were worse by 3.30 points (95% CI: −6.54 to −0.06, *p* = .05) at three months (Table 2) and by 2.46 points (95% CI: −5.72 to 0.79, *p* = .14) at six months (Supplementary Table S5) in the Take Charge group compared to control group. The GDS scores were significantly higher in the Take Charge group compared to control group at six months using both models (Supplementary Table S5).

**Table 2.** Summary of primary and secondary outcomes and adjusted mean differences at three months.

Outcome measures	Take charge		Control		Model 1 (three months)	Model 2 (three months)
	TC_Baseline Mean (*SD*)	TC_3m Mean (*SD*)	C_Baseline Mean (*SD*))	C_3m Mean (*SD*)	MD (95% CI), *p*-value	Adjusted MD (95% CI), *p*-value
**Primary outcome**						
**Standardized PCS score**	41.6 (12.0)	43.4 (12.0)	42.6 (11.6)	43.2 (11.0)	−0.53 (−4.23, 3.17), *p* = .78	−0.78 (−4.48, 2.91), *p* = .68
**Secondary outcomes**						
**Standardized MCS score**	49.0 (9.7)	49.2 (10.4)	49.6 (11.0)	53.2 (9.4)	−3.75 (−7.03, −0.47), *p* = .02*	−3.30 (−6.54, −0.06), *p* = .05*
**AHS total hope score**	47.8 (7.9)	48.2 (9.1)	49.4 (8.0)	50.8 (9.2)	−2.39 (−5.18, 0.41), *p* = .09	−2.12 (−4.91, 0.67), *p* = .14
**FAI total activity score**	24.7 (8.1)	23.3 (8.7)	26.6 (6.4)	26.0 (7.3)	−3.22 (−5.70, −0.74), *p* = .01*	−3.44 (−5.69, −1.19), *p* = .003*
**GDS total depression score**	4.2 (3.3)	3.9 (3.0)	4.0 (3.0)	3.2 (2.4)	0.77 (−0.21, 1.75), *p* = .12	0.70 (−0.27, 1.67), *p* = .16

Abbreviations: *SD*, standard deviation; TC, Take Charge; C, control; 3 m, three months; MD, mean difference; PCS, Short Form-36 physical component summary; MCS, Short Form-36 mental component summary; AHS, adult hope scale; FAI, Frenchay activities index; GDS, geriatric depression scale.

* Significant difference.

*Notes*. The mean differences were calculated using Linear Mixed model. Model 1 considered treatment (Take Charge vs control), time, and time & treatment interaction only. Model 2 was extension of Model 1, considered treatment, time, treatment & time interactions and adjusted for covariates such as age (centred at own mean), gender, living situation, diagnosis, and level of cognitive impairment.

Table 3 shows the overall intervention effects. The Take Charge intervention had no significant impact on SF-36 PCS scores compared to the control group (adjusted mean difference: −0.97; 95% CI: −4.60 to 2.66; *p* = .60), with no significant improvement at three or six months. Within-participant PCS score variability was 9.94 points (ICC 0.73). No significant interaction between intervention and time was observed (*p* = .59; Supplementary Table S6). SF-36 MCS scores improved at three months (MD: 3.49; 95% CI: 1.30–5.67; *p* < .01) and six months (MD: 2.81; 95% CI: 0.63–4.99; *p* = .01). GDS scores also showed small improvements at both time points (−0.63; 95% CI: −1.18 to −0.08; *p* = .03 and −.68; 95% CI: −1.24 to −0.12; *p* = .02, respectively).

**Table 3.** Overall Adjusted Linear Mixed effect model estimates for primary and secondary outcome measures.

	PCS score estimates, MD (β) [95% CI], *p*-value	MCS score estimates, MD (β) [95% CI], *p*-value	AHS score estimates, MD (β) [95% CI], *p*-value	FAI score estimates, MD (β) [95% CI], *p*-value	GDS score estimates, MD (β) [95% CI], *p*-value
***Fixed effect (between participant effect)***					
**Treatment effect: control (ref)**					
**Take Charge**	−0.97 [−4.60, 2.66], *p* = .60	−0.00 [−3.16, 3.16], *p* = 1.00	−1.49 [−4.24, 1.25], *p* = .29	−2.16 [−4.36, 0.04], *p* = .054	0.21 [−0.75, 1.16], *p* = .67
**Time effect: baseline (ref)**					
**Three month**	0.49 [−1.44, 2.43], *p* = .62	3.49 [1.30,5.67], *p* < .01	1.10 [−0.59, 2.79], *p* = .20	−0.83 [−2.05, 0.39], *p* = .18	−0.63 [−1.18, −0.08], *p* = .03
**Six month**	1.40 [−0.54, 3.33], *p* = .16	2.81 [0.63, 4.99], *p* = .01	0.01 [−1.70, 1.73], *p* = .99	−2.21 [−3.44, −0.98], *p* < .01	−0.68 [−1.24, −0.12], *p* = .02
**Treatment × time interaction**					
**Take Charge × three months**	0.19 [−2.58, 2.96], *p* = .90	−3.30 [−6.42, −0.18], *p* = .04	−0.63 [−3.05, 1.80], *p* = .61	−1.28 [−3.02, 0.456], *p* = .15	0.49 [−0.28, 1.27], *p* = .21
**Take Charge × six months**	−1.09 [−3.86, 1.69], *p* = .44	−2.46 [−5.60, 0.67], *p* = .12	0.74 [−1.73, 3.20], *p* = .56	−0.14 [−1.88, 16.0], *p* = .88	0.88 [0.09, 1.67], *p* = .03
***Random effect (within participant effect)***					
**SD (ICC)**	9.94 (0.73)	7.43 (0.54)	6.97 (0.63)	5.94 (0.71)	2.54 (0.68)

Notes. The above results for mixed model considered treatment (Take Charge & control), time (0 months, 3 months, 6 months), and time & treatment interaction adjusted for age (centered at mean), gender (male/female), Diagnosis (dementia/MCI), living situation (living alone/with family), and level of cognitive impairment (MMSE<=23 / MMSE >23).

Abbreviations. MD, mean difference; β, beta coefficient of change; CI, confidence interval; PCS, Short Form-36 physical component summary; MCS, Short Form-36 mental component summary; AHS, adult hope scale; FAI, Frenchay activities index; GDS, geriatric depression scale; *SD*, standard deviation; ICC, intraclass correlation coefficient.

### Subgroup analysis

Supplementary Table S7 shows no significant differences in health-related PCS, MCS, AHS, FAI, or GDS scores between participants with dementia and MCI at three months. The interaction test confirmed no heterogeneity in treatment effect by diagnosis (*p* = .78 for PCS). The pre-planned analysis of baseline characteristics found no significant PCS score differences by gender, living situation, or cognitive impairment level (Supplementary Table S8).

At six months, the RUD questionnaire showed no significant differences in caregiving time (*p* = .81), care partner productivity loss (*p* = .46), or health resource use (*p* = .13) (Supplementary Table S9). Evaluation results (Supplementary Table S10) indicate that most participants (86.4%) found the project well-explained, 72.8% reported some benefit, and 63.9% felt more hopeful about their health. However, additional comments suggest survey responses may not fully capture the project’s impact.

### Qualitative findings

Nine Take Charge participants participated in one-off interviews about their experience with the intervention, five of whom included their care partners. Three key themes emerged: “Building self-awareness,” “Is the intervention approach suitable for everybody?”, and “I look at things a little bit more positive now.” These themes are discussed in subsequent text, with pseudonyms used to protect participant anonymity and added context in [brackets] for clarity. Table 4 includes quotes to support the themes.

**Table 4.** Quotes to support interview themes.

Theme	Quote
**Building self-awareness**	Well, there’s lots of things that I put off doing or don’t do, but it was an opportunity to think about those. (Vicki). I’ve learned a couple of things and I have to think and concentrate on making sure that I do try and follow up on what I’ve said. (Jack)
**Is the intervention approach suitable for everybody?**	… was great at the time. But straight afterwards, it meant [nothing to the participant]… I think some people would get a lot more out of it if [they took part] at an earlier stage. (James, carer) I probably didn’t take in… I don’t really know why I didn’t but, uh… probably ‘cause I’m big-headed or something. (Harry) I wasn’t sure with my attitude to things and how I’m lazy and don’t sorta fill out too much stuff, whether that would maybe be a help or not. But if it is, good. (Jack)
**I look at things a little bit more positive now**	When you retire, you don’t sort of stop and think about a lot of things and, uh, in sitting down and talking with, uh, [with the Take Charge interventionist], the thing, we sort of stop and think, “Well, hm, we should’ve been doing that,” or, “We could’ve done that,” or whatever, you know? (Mary) I could talk about it [dementia] with other people… I’m comfortable talking about it with them now when originally [I wasn’t]. (Tom) I’ve learned a couple of things and I have to think and concentrate on making sure that I do try and follow up on what I’ve said. (Jack)

#### Building self-awareness

Participating in Take Charge was seen as a way of gaining “more insight about the situation, you know, like how I’m going” (Donna) following their recent diagnosis. It provided participants with an opportunity to reflect on how they approached different situations or tasks in life and build self-awareness (Table 4). The intervention was also seen as a way of empowering the participants to take ownership of their planned actions and follow through on intentions (Table 4). One participant described that Take Charge had taught her to make the most of the days to come.

I think um, in general plus if um, maybe with my diagnosis, I would, I’ve thought about it and I want to make sure that I get the best out of what I do nowadays. (Donna)

#### Is the intervention approach suitable for everybody?

A recurring theme in about half of the interviews was whether the intervention approach was suitable for everyone. For instance, one carer noted that the participant’s dementia might have been too advanced for them to fully engage with or benefit from Take Charge (Table 4).

Take Charge encourages participants to actively take ownership of what lies ahead, including setting potential goals. However, this was not acted upon by all participants.

I don’t need goals because everyday getting up is a new day and I just, whatever I have to do on that day, I do. (Elsa)

One participant talked about whether he understood the purpose of the intervention and took in what it had to offer (Table 4). Whereas others mentioned that they likely did not have the best attitude toward taking part in the Take Charge activities (Table 4).

#### I look at things a little bit more positive now

About half of the participants reported actively engaging with the Take Charge activities and found the experience valuable. They described it as an opportunity to reflect on their lives since diagnosis, which they appreciated. One participant, for example, described the program as a chance to pause, reset, and consider what she could have done or could do differently (Table 4). Another participant described that Take Charge helped him feel more comfortable in terms of talking about his diagnosis (Table 4).

It also appeared that, since participating in Take Charge, there had been a shift in attitudes (Table 4). Some participants even described that they were now keen to make the best of the days to come.

I look at things a little bit more positive now. If that makes sense… I’ve thought about [my diagnosis] and I want to make sure that I get the best out of what I do nowadays. (Donna)

## Discussion

This study evaluated the Take Charge intervention for people with MCI or mild dementia, compared to post-diagnostic lifestyle education. Both groups received equal interaction with a health professional. While no significant effect was found on physical health-related QoL (SF-36 PCS), small but significant improvements were observed in mood (GDS) and mental health-related QoL (SF-36 MCS) for the overall study cohort, though these may not be clinically meaningful (Vinkers et al., 2004).

Participants found the intervention engaging, with interviews suggesting it fostered self-awareness. However, many struggled to remember or follow through on goals, a challenge also noted anecdotally by families and in team discussions. Similar findings have been observed in other dementia studies. A process evaluation of a self-directed exercise and functional activity intervention for people with early-stage dementia found that while participants valued the intervention, cognitive impairment limited independent engagement and program continuity (Di Lorito et al., 2023). Another intervention designed to promote independence and self-management in people with dementia, despite being tailored to participants’ needs, did not improve QoL at eight months (Mountain et al., 2022). A decline in executive function, an early symptom of dementia (Allain et al., 2013), may limit the effectiveness of such self-directed programs and it is possible that we underestimated the extent to which a high level of executive functioning was required to benefit from Take Charge. Further research is needed to whether self-directed interventions can be adapted for this population. Potential adaptations could include visual and communication supports, creating and using tracking tools such as calendars or other progress charts, and involving care partners (Jogie et al., 2025; Parsons & Parsons, 2012).

People with dementia and their care partners have told us that this is the type of program that they want (focused on hope and quality of life and the values and priorities of the person with dementia) (Dementia Australia, 2019). However, for some of our participants, demonstrating self-determination was difficult. It is possible that the intervention was too nuanced and more visits, more care partner participation and a more explicit approach to identifying goals and determining strategies to achieve these goals is required. Care partners were not required to be involved in the Take Charge intervention, however family and friends are usually included in psychoeducational interventions for people with dementia. Studies from the United Kingdom have found significant improvements in goal attainment for people with dementia compared with routine care when family care partners were engaged in both intervention delivery and evaluation (Clare et al., 2019; Cooper et al., 2024). One program focused on goal-oriented cognitive rehabilitation (Clare et al., 2019), while the other focused on self-identified concerns using a manualized program approach with nonclinical facilitators (Cooper et al., 2024). Both involved multiple components and regular follow-ups, reinforcing the importance of family support in dementia care. Research consistently shows that engaging care partners in dementia care leads to better outcomes (Laver et al., 2017; Teahan et al., 2020). The Goal Attainment Scale may also be a more appropriate measure for evaluating the success of interventions involving goal setting.

Take Charge was initially developed and shown to be effective for individuals who had experienced stroke, particularly for physical health-related QoL, independence and advanced activities of daily living. While stroke is a life-changing event, the person is expected to experience a degree of natural recovery toward their previous level of function. A diagnosis of cognitive impairment or dementia leads to different trajectory in function and independence compared to stroke. Cognitive impairment and dementia are progressive conditions without a sudden precipitating neurological event. Given the progressive nature of dementia, the benefit of intervention may be more difficult to detect. In people with dementia, maintaining function and quality of life or slowing down the progression of decline can be a worthwhile outcome of interest in studies evaluating the impact of self-directed programs.

Another possible explanation for our findings is the similarity between intervention and control groups. Unlike previous Take Charge studies, where controls received only a pamphlet, our control group had equivalent health professional interaction and post-diagnostic education, potentially diluting differences. Supporting this, both groups showed unexpected improvements in PCS over six months (Supplementary Table S3), and participant feedback indicated benefits across both groups (Supplementary Table S7).

This is the fourth randomized trial of the Take Charge, previously tested in stroke (Fu et al., 2020; Harwood et al., 2012) and COPD (Levack et al., 2023) populations. While the intervention shows promise as a simple, low-cost approach to person-centered care, its benefits for people with MCI or mild dementia may be more limited. Nonetheless, the growing evidence base warrants further attention from clinicians, funders, and researchers.

### Strengths and limitations

A key strength of this study is that Take Charge addressed participants’ priorities, fostering ownership and future planning, enhancing its transferability. The program is adaptable, allowing for cultural sensitivity by incorporating individual life experiences, values, and goals, making it responsive to diverse cultural needs and effective in minority populations (Harwood et al., 2012). Recruitment from a hospital-based setting supports the generalizability of the findings. In addition, the high consent and low drop-out rates suggest openness to interventions in this population. A limitation is that this is a small study, and participants were recruited from one city in Australia. The use of the same health professional for both Take Charge and control interventions may have introduced contamination. Consumer involvement was also limited, with only one representative (a spouse of someone with dementia) engaged, and additional perspectives from people living with dementia or MCI would have enriched the study’s depth and relevance.

## Conclusion

Interventions that are guided by self-determination and intrinsic motivation can foster positive outcomes for individuals facing life-changing health events, such as stroke. Our study suggests that such interventions can also be well-received by those newly diagnosed with MCI or mild dementia. Although the Take Charge intervention did not significantly improve physical health-related QoL compared to a wait-list control with lifestyle education, we observed improvements in mental health outcomes, including mood, for the entire study group. For individuals actively participating in the intervention, Take Charge helped build self-awareness and encouraged a more positive outlook. Further research is needed to determine whether such self-directed interventions can be adapted for this population and to identify which individuals with cognitive impairment and dementia are likely to benefit.

## Supplementary Material

gnaf280_Supplementary_Data

## Data Availability

The data used in this study are not publicly available due to conditions granted the human research ethics committee. Intervention manuals are publicly available online https://www.mrinz.ac.nz/take-charge-rehabilitation-resources. The study was preregistered with the Australian New Zealand Clinical Trials Registry [Registration number: ACTRN12621000282886].
