# Supplementary material for: “Taking Charge” after a diagnosis of cognitive impairment or dementia: a randomized controlled trial
Source: Gerontologist. 2025 Nov 24;65(12):gnaf280. doi: 10.1093/geront/gnaf280 (PMC12742850; doi:10.1093/geront/gnaf280)
Supplement: gnaf280_Supplementary_Data [file gnaf280_supplementary_data.pdf]

'Taking Charge' after a diagnosis of cognitive impairment or dementia: A randomised controlled trial

Authors: Rahja Miia, McNaughton Harry, Crotty Maria, Gulyani Aarti, Xu Qunyan, Davies Owen, Santosaputri Elita and Laver Kate

Table S1. Description of outcome measures used

Outcome assessment	Description	Scoring methods used in this study
Short Form 36 items (SF-36)(Hays et al., 1993)	The SF-36 is a set of generic, coherent, and easily administered quality-of-life measures. These measures rely upon patient self-reporting and are widely used for routine monitoring and assessment of care outcomes in adult patients. All items are scored so that a high score defines a more favourable health state. Each item is scored on a 0 to 100 range so that the lowest and highest possible scores are 0 and 100, respectively.	A three-step process was used as follows:<br>1) calculating eight (see below) raw subscale scores from the SF-36;<br>2) calculating the raw physical component summary (PCS) and mental component summary (MCS) scores; and<br>3) calculating the standardised PCS and MCS scores.<br>The eight raw subscales were physical functioning (PF), role—physical (RP), bodily pain (BP), general health (GH), vitality (VT), social functioning (SF), role—emotional (RE) and mental health (MH). Scales PF, RP, BP and GH contribute to the physical health component and scales VT, SF, RE and MH contribute to mental health component scores. The subscale scores were calculated via a two-step process: 1) the original response to an individual item was recoded to the 100-point scale, with a higher score indicating better health state for all 36 items (Ware, Snoww, et al., 1993). 2) Second, the items in the same scare were averages together to create the 8 scale score when 50% or more items recorded a non-missing response (Ware, Snoww, et al., 1993).<br>The raw PCS and MCS scores were calculated using the steps outlined by Ware and colleagues (Ware, Ma, et al., 1993) using the SF-36 norms obtained from the 1995 Australian general population norms (Australian Bereau of Statistics, 1995). First, each of the eight raw subscale scores were transformed to normalized z-scores by using the corresponding subscale mean and standard deviation derived from the 1995 Australian general population. Secondly, raw PCS and MCS score was calculated as the sum of the product of each z subscale score and their corresponding PCS and weights respectively (Australian Bereau of Statistics, 1995). Standardized PCS and standardized MCS scores were calculated by multiplying raw PCS/MCS scores with 10 and adding 50 (Ware, Ma, et al., 1993).
Frenchay Activities Index	The FAI was developed specifically for measuring disability in stroke patients and is a useful instrument to assess	The total FAI score was calculated as the sum of all 15 items as per Wade and colleagues (Wade et al., 1985) where the authors proposed

(FAI) (Schuling et al., 1993).	functional status. It was tested in a group of stroke patients and a group of unselected subjects aged 65 or older. The tool has shown sufficient reliability scores (range of Cronbach's alpha-coefficients, 0.78 to 0.87) and construct validity has been supported by meaningful correlations between the Frenchay Activities Index and scores on the Barthel Index and Sickness Impact Profile.<br>The FAI contains 15 items that can be separated into 3 subscales; Domestic chores, Leisure/work and Outdoor activities and the frequency with which each item or activity is undertaken over the past 3 or 6 months (depending on the nature of the activity).	the 0 to 3 point scoring algorithm for each item. Score 0 suggests lowest level of activity. Three subscale scores were also calculated. Specifically, the domestic subscale score was the sum of item 1 to 5; the work/leisure subscale score was the sum of items 7, 9, 11, 13, 15; and outdoor subscale score the sum of items 6, 8, 10, 12, 14. While we investigated the subscale scores in this study, we acknowledge that Wade and colleagues (Wade et al., 1985) have questioned the appropriateness of such grouping.
Adult Hope Scale (AHS) (Snyder et al., 1991)	The AHS is a 12-item measure of a respondent's level of hope. It is divided into two subscales of hope (1) Agency (i.e., goal-directed energy) and (2) Pathways (i.e., planning to accomplish goals). Of the 12 items, 4 make up the Agency subscale and 4 make up the Pathways subscale. Each item is answered using an 8-point Likert-type scale ranging from Definitely False to Definitely True. Researchers can examine results at the subscale level or combine the two subscales to create a total hope score. The higher the score the higher agency/ pathways thinking of hope is present.	The total AHS score was calculated as the sum of item 1, 2, 4, 6, 8, 9, 10 & 12. The agency subscale score was the sum of item 2, 9, 10, 12; and the pathway subscale score was the sum of item 1, 4, 6, 8 (Snyder et al., 1997). All items are measured on an 8-point scale, with 1 indicating "Definitely false" and 8 "Definitely true", therefore the lowest possible total hope score is 8, and highest 64. Participants were classified as "not hopeful" if their total hope score was between [8, 40), "hopeful" [40-48), "moderately hopeful" [48,56), and "high hope" if their total hope score was within [56,64].
Geriatric depression scale (GDS) (Sheikh & Yesavage, 1986)	The 15-item short version of the GDS is a validated self-report measure of depression in older adults. Users respond in a "Yes/No" format. A validation study conducted by Sheikh and Yesavage (Sheikh & Yesavage, 1986) found that the GDS-S was compared to the GDS-L in differentiating depressed from non-depressed patients. Both measures were successful in classifying the 2 populations accurately with a reported correlation of r =0.84, p<0.001.	The GDS has fifteen (15) questions, with provided responses "Yes", or "No". A response of "Yes" to questions 2,3,4,6,8,9,10,21,14,15 and "no" to questions 1,5,7,11, and 13 indicate depression. One point is scored for each one selected. The sum of all points was taken to be the GDS depression score. Participants with a GDS depression score greater than 5 points were classified as having depression, and those with a GDS depression score lower than/equal to 5 as not having depression (Sheikh & Yesavage, 1986).
Evaluation of project (3 months only)	The following 6 questions were posed in the program evaluation.<br>1. Was the project clearly explained to you from the time we first contacted you?<br>2. Overall, how much do you think you benefitted from participation in this project?<br>3. How much did participation in this project help you feel more hopeful about your future health?	The response options for questions 1 and 6 were "No", "Yes", "Unknown".<br>The response options for questions 2-4 were "Not at all", "Some", "A great deal", "Unknown", "Declined to answer".<br>The participants were also asked a question: Do you have any further comments that you would like to make about the program?

	4. How much did participation in the project help enhance your ability to understand how to achieve a healthy lifestyle?<br>5. Did the project require too much work or effort?<br>6. Would you recommend this project to others in similar situations?	
Short version of the Resource Utilisation in Dementia (RUD); RUD-Lite (Wimo et al., 2010; Wimo & Winblad, 2003)	The revised (Lite) version of the RUD instrument has been validated for assessing care accessed in community dwelling people with dementia in Sweden.<br>The purpose of the RUD-Lite instrument was to collect data on resource use to calculate costs of patient care (healthcare resource utilisation) and care partner time in Dementia.	Hypothesis testing of the effect of Take Charge intervention relative to the control group on health service utilisation was measured at 6 months post baseline with care partners of participants.<br>In Rud-Lite questionnaire, total number of hours spent care giving in the last 30 days was calculated as the sum of hours spent assisting activities of daily living (ADLs), activities of instrumental activities of daily living (IADL) and supervising the older person with mild cognitive impairment. Total no. of visits that use health resources in the last 30 days was calculated as sum of visits to health professionals, by nurse and home help, and hospital and emergency department (ED) admissions.

Table S2. Missing Data Table

Primary and Secondary Outcomes	Observations	Missing at Baseline			Missing at 3 Months		
		Included (n)	Missing (n)	Missing %	Included (n)	Missing (n)	Missing %
**SF-36 Subscales**							
Physical Function (PF)	160	160	0	0.0	149	11	6.9
Role Physics (RP)	160	158	2	1.3	149	11	6.9
Bodily Pain (BP)	160	159	1	0.6	150	10	6.3
General Health (GH)	160	158	2	1.3	150	10	6.3
Vitality (VT)	160	157	3	1.9	149	11	6.9
Social Functioning (SF)	160	159	1	0.6	148	12	7.5
Role Emotional (RE)	160	158	2	1.3	149	11	6.9
Mental Health (MH)	160	157	3	1.9	149	11	6.9
**SF-36 Standardised scales**							
Standardised PCS score	160	157	3	1.9	146	14	8.8
Standardised MCS score	160	157	3	1.9	146	14	8.8
**Other outcomes measures**							
AHS agency subscale score	160	157	3	1.9	150	10	6.3
AHS pathway subscale score	160	157	3	1.9	150	10	6.3
AHS total score	160	157	3	1.9	150	10	6.3
FAI domestic subscale score	160	160	0	0.0	145	15	9.4
FAI leisure/work subscale score	160	160	0	0.0	146	14	8.8
FAI outdoor subscale score	160	160	0	0.0	145	15	9.4
FAI total score	160	160	0	0.0	145	15	9.4
GDS total score	160	160	0	0.0	149	11	6.9

Abbreviations used: SF-36, Short Form 36 items; PCS, Short Form-36 physical component summary; MCS, Short Form-36 mental component summary; AHS, adult hope scale; FAI, Frenchay activities index; GDS, geriatric depression scale.

Table S3. Baseline participant and carer characteristics by diagnosis

Characteristics	Dementia	MCI	p-value
Number of participants	93	67	
Age of participant (years), mean (SD)	79.9 (7.5)	79.1 (6.9)	0.46
**Participant related**			
Gender of participant, n (%)			0.59
Female	46 (49.5%)	36 (53.7%)	
Male	47 (50.5%)	31 (46.3%)	
MMSE score, mean (SD)	22.9 (2.8)	25.7 (2.9)	<0.001*
Living situation, n(%)			0.17
Alone	19 (20.4%)	20 (29.9%)	
With family	74 (79.6%)	47 (70.1%)	
**Outcome scores at baseline, mean (SD)**			
Short Form 36 (SF-36)			
SF36 Standardized PCS score	40.9 (11.8)	43.7 (11.6)	0.14
SF36 Standardized MCS score	50.0 (10.6)	48.3 (10.0)	0.30
SF36 PF score	57.8 (29.0)	64.6 (27.1)	0.13
SF36 RP score	59.3 (44.0)	64.9 (41.3)	0.42
SF36 BP score	72.9 (26.5)	69.0 (24.9)	0.34
Raw GH score	56.6 (19.2)	63.7 (19.3)	0.023*
SF36 VT score	55.5 (20.9)	54.6 (19.7)	0.79
SF36 SF score	78.9 (24.2)	77.8 (20.9)	0.76
SF36 RE score	78.0 (34.1)	69.7 (37.9)	0.15
SF36 MH score	73.4 (18.3)	73.5 (16.6)	0.97
AHS total hope score	48.0 (8.8)	49.4 (6.5)	0.25
AHS agency subscale score	24.3 (5.1)	25.6 (3.2)	0.058
AHS pathway subscale score	23.7 (4.5)	23.8 (4.1)	0.86
Adult Hope Scale (AHS) hope level, n (%)			0.15
Not hopeful	11 (12.2%)	5 (7.5%)	
Hopeful	29 (32.2%)	18 (26.9%)	
Moderately hopeful	31 (34.4%)	35 (52.2%)	
Highly hopeful	19 (21.1%)	9 (13.4%)	
FAI total score	24.2 (7.5)	27.8 (6.5)	0.002*
FAI domestic subscale score	9.6 (3.9)	10.4 (3.7)	0.18
FAI leisure/work subscale score	6.4 (2.8)	7.5 (1.9)	0.007*
FAI outdoor subscale score	8.1 (3.6)	9.8 (3.6)	0.003*
GDS score	4.5 (3.4)	3.6 (2.8)	0.075
GDS depression status, n (%)			0.31
No depression	67 (72.0%)	53 (79.1%)	
Depression (GDS score>5)	26 (28.0%)	14 (20.9%)	
**Carer related**			
Gender of carer, n (%)			0.77
Female	54 (66.7%)	36 (64.3%)	
Male	27 (33.3%)	20 (35.7%)	
Relationship to patient, n (%)			
Spouse/partner	57 (70.4%)	35 (62.5%)	
Sibling	0 (0.0%)	1 (1.8%)	
Child	22 (27.2%)	17 (30.4%)	
Friend	0 (0.0%)	1 (1.8%)	
Other	2 (2.5%)	2 (3.6%)	

Notes: * indicates significant difference. P values were obtained using chi-squared test for categorical variables or two-sample t-test for continuous variables between the dementia and MCI groups. Abbreviations used: MCI, mild cognitive impairment; MMSE, mini mental status examination; SF36, short form 36 questions; PF, physical functioning; RP, role—physical; BP, bodily pain; GH, general health; VT, vitality; SF, social functioning; RE, role—emotional; MH, mental health; PCS, physical component summary; MCS, mental component summary; AHS, adult hope scale; FAI, Frenchay activities index; GDS, geriatric depression scale.

Table S4. Summary statistics on (sub)scales for all outcome measures at baseline, 3- and 6-months

	Take Charge			Control		
**Subscale measures**	**Baseline Mean (SD)**	**3-months Mean (SD)**	**6-months Mean (SD)**	**Baseline Mean (SD)**	**3-months Mean (SD)**	**6-months Mean (SD)**
SF36 PF	59.0 (30.4)	67.3 (27.7)	64.8 (29.0)	62.3 (26.2)	67.3 (26.5)	67.0 (26.4)
SF36 RP	62.2 (43.2)	61.6 (41.7)	61.1 (39.8)	61.2 (42.8)	62.5 (36.6)	67.2 (37.2)
SF36 BP	72.2 (25.8)	75.1 (26.8)	74.9 (29.3)	70.3 (26.0)	76.9 (25.0)	76.8 (24.8)
SF36 GH	56.7 (19.1)	56.4 (23.9)	58.4 (24.6)	62.5 (19.6)	62.0 (23.3)	63.8 (21.5)
SF36 VT	53.6 (21.0)	51.8 (26.0)	51.0 (25.3)	56.6 (19.6)	57.3 (21.7)	57.5 (23.7)
SF36 SF	79.0 (22.6)	80.0 (25.9)	81.2 (28.1)	78.0 (23.1)	88.7 (18.4)	85.6 (22.3)
SF36 RE	75.2 (35.8)	82.2 (33.4)	81.0 (34.3)	73.8 (36.2)	82.0 (31.9)	85.1 (31.8)
SF36 MH	72.6 (17.9)	74.4 (18.3)	75.6 (19.9)	74.2 (17.3)	81.1 (15.1)	79.3 (15.4)
Standardised PCS	41.6 (12.0)	43.4 (12.0)	42.9 (12.2)	42.6 (11.6)	43.2 (11.0)	43.9 (10.8)
Standardised MCS	49.0 (9.7)	49.2 (10.4)	49.8 (11.1)	49.6 (11.0)	53.2 (9.4)	52.3 (9.8)
AHS agency subscale	24.6 (4.1)	24.6 (5.0)	24.9 (5.7)	25.1 (4.8)	25.3 (5.4)	25.0 (5.2)
AHS pathway subscale	23.2 (4.6)	23.5 (5.1)	24.0 (5.1)	24.4 (4.0)	25.5 (5.0)	24.6 (4.5)
AHS total hope	47.8 (7.9)	48.2 (9.1)	48.9 (9.8)	49.4 (8.0)	50.8 (9.2)	49.7 (8.6)
FAI domestic subscale	9.6 (4.0)	8.9 (4.5)	9.2 (4.6)	10.3 (3.5)	10.2 (3.7)	9.3 (4.0)
FAI leisure/work subscale	6.8 (2.3)	5.8 (2.9)	5.6 (2.6)	6.9 (2.8)	6.4 (2.6)	6.2 (2.4)
FAI outdoor subscale	8.3 (4.1)	8.6 (3.8)	8.4 (3.9)	9.4 (3.2)	9.4 (3.8)	8.9 (3.6)
FAI total	24.7 (8.1)	23.3 (8.7)	23.1 (8.6)	26.6 (6.4)	26.0 (7.3)	24.4 (7.5)
GDS total depression	4.2 (3.3)	3.9 (3.0)	4.0 (3.6)	4.0 (3.0)	3.2 (2.4)	3.1 (2.7)

Abbreviations used: SD, standard deviation; SF36, short form 36 questions; PF, physical functioning; RP, role—physical; BP, bodily pain; GH, general health; VT, vitality; SF, social functioning; RE, role—emotional; MH, mental health; PCS, physical component summary; MCS, mental component summary; AHS, adult hope scale; FAI, Frenchay activities index; GDS, geriatric depression scale.

Supplementary file. 'Taking Charge' after a diagnosis of cognitive impairment or dementia: A randomised controlled trial

Table S5. Take Charge intervention effect on primary and secondary outcomes at 6-months

**Outcome**	**Linear Mixed Model**	**Mean difference at 6-months**	**P_value**
Standardised PCS score	Model 1	-1.79 (-5.50, 1.92)	0.345
	Model 2	-2.06 (-5.77, 1.65)	0.276
Standardised MCS score	Model 1	-2.96 (-6.26, 0.33)	0.078
	Model 2	-2.46 (-5.72, 0.79)	0.138
AHS total score	Model 1	-1.04 (-3.87, 1.79)	0.472
	Model 2	-0.76 (-3.59, 2.07)	0.598
FAI total score	Model 1	-2.10 (-4.57, 0.38)	0.097
	Model 2	-2.30 (-4.55, -0.04)	0.046*
GDS (depression) score	Model 1	1.16 (0.17, 2.15)	0.022*
	Model 2	1.09 (0.10, 2.07)	0.031*

Note: Model 1 considered treatment (take-charge vs control), time (baseline, 3 months, and 6 months),) and time & treatment interaction only. Model 2 was adjusted for covariates and considered treatment, time, treatment x time interactions, age (centred at own mean), gender, living situation, diagnosis, and level of cognitive impairment.

Abbreviations used: PCS, physical component summary; MCS, mental component summary; AHS, adult hope scale; FAI, Frenchay Activities Index; GDS, Geriatric Depression Scale.

Table S6. Interaction test: Treatment X time from Repeated Measure ANOVA

Ho: No significant treatment by time interaction<br>*Ha: There is significant treatment by time interaction*		
**Outcome**	**F-statistics**	**P-value**
Standardised PCS Score	0.54	0.586
Standardised MCS Score	2.74	0.066
Total FAI Score	1.34	0.265
Total AHS Score	0.45	0.63
GDS Score	2.86	0.059
Notes: Ho, Null hypothesis; Ha, Alternative hypothesis.<br>Abbreviations used: PCS, physical component summary; MCS, mental component summary; AHS, adult hope scale; FAI, Frenchay Activities Index; GDS, Geriatric Depression Scale.		

Supplementary file. 'Taking Charge' after a diagnosis of cognitive impairment or dementia: A randomised controlled trial

Table S7. Subgroup Analysis by randomisation stratification factor – Diagnosis: Mean differences in scores between the Take-charge and control group at 3 months

Outcome Measures	Diagnosis	Mean difference (95% CI)[a] at 3-months	P value for the interaction [b]
Standardised PCS score	MCI	-1.06 (-6.42, 4.31), p=0.70	0.78
	Dementia	-0.09 (-4.76, 4.59), p=0.97	
Standardised MCS score	MCI	-3.81 (-8.38, 0.76), p=0.10	0.97
	Dementia	-3.70 (-7.75, 0.35), p=0.07	
AHS total score	MCI	-2.06 (-6.05, 1.92), p=0.31	0.83
	Dementia	-2.58 (-6.07, 0.90), p=0.15	
FAI total score	MCI	-1.77 (-5.26, 1.72), p=0.32	0.28
	Dementia	-4.14 (-7.18, -1.11), p=0.01	
GDS total score	MCI	0.63 (-0.76, 2.02), p=0.46	0.81
	Dementia	0.84 (-0.38, 2.05), p=0.18	

Notes:
a. Mean differences (95%CI) were calculated by using treatment (Take-charge vs control), time (baseline, 3m, 6m), diagnosis (MCI vs dementia), treatment into time interaction and treatment into diagnosis in a mixed model.
b. Interaction was tested for Mean difference of outcome measure for people diagnosed with MCI in Take-charge group at 3-month = Mean difference of outcome measure for people diagnosed with Dementia in Take-charge group at 3-month.

Abbreviations used: PCS, physical component summary; MCS, mental component summary; AHS, adult hope scale; FAI, Frenchay Activities Index; GDS, Geriatric Depression Scale.

Table S8. Test of effect modification by gender, diagnosis, living situation and level of cognitive impairments on PCS score over time

Interactions: treatment (Take Charge/control) X time (baseline, 3-months, 6-months) X effect modifiers (gender, diagnosis, living situation and level of cognitive impairment)

Effect modifier	Chi-square	P-value for Interaction	Adjusted P-value (Bonferroni correction)
**Primary Outcome (PCS score)**			
Cognitive impairment	0.64	0.72	1.00
Gender	1.88	0.39	0.78
Diagnosis	0.35	0.84	1.00
Living situation	2.94	0.23	0.46

Notes:
a. Levels of potential effect modifiers are gender (male/female), diagnosis (dementia/MCI) living situation (living alone/with family and level of cognitive impairment (MMSE<=23 vs. MMSE >23).
b. Coefficients were calculated using Model 2 including treatment into time into modifier interaction.
c. Test is significant if P-value <= 0.01.

Supplementary file. 'Taking Charge' after a diagnosis of cognitive impairment or dementia: A randomised controlled trial

Table S9. Carers data and health service utilisation using RUD-Lite questionnaire at 6-months

**Characteristics**	**Total**	**Take Charge**	**Control**	**P-value**
**Total carers, n**	**137**	**65**	**72**	
Percentage of care, n (%)				0.41
1-20%	4 (2.9%)	2 (3.1%)	2 (2.8%)	
21-40%	4 (2.9%)	1 (1.5%)	3 (4.2%)	
41-60%	6 (4.4%)	5 (7.7%)	1 (1.4%)	
61-80%	17 (12.4%)	8 (12.3%)	9 (12.5%)	
81-100%	106 (77.4%)	49 (75.4%)	57 (79.2%)	
Hours spent care giving in the last 30 days, mean (SD)	54.2 (90.8)	55.9 (88.4)	52.5 (93.8)	0.81
Hours spent assisting ADLs in the last 30 days, mean (SD)	8.2 (27.4)	10.8 (29.6)	5.7 (25.2)	0.28
Hours spent assisting IADLs in the last 30 days, mean (SD)	45.6 (61.5)	44.8 (51.1)	46.5 (70.5)	0.88
Hours spent supervising in the last 30 days, mean (SD)	12.5 (48.8)	14.0 (48.7)	11.0 (49.3)	0.73
Worked for pay or not in the last 30 days, n (%)				0.46
Yes	34 (24.8%)	18 (27.7%)	16 (22.2%)	
No	103 (75.2%)	47 (72.3%)	56 (77.8%)	
Number of workdays affected by caring duty in the last 30 days, median (IQR)	0.5 (0-2)	0.5 (0-2)	0.75 (0-2)	1.00
Number of visits that use health resources in the last 30 days, median (IQR)	3 (1-5.5)	2 (0.5-5.5)	3 (1-5.5)	0.13
Number of hospital & ED admissions in the last 30 days, median (IQR)	0 (0-0)	0 (0-0)	0 (0-0)	0.98
Number of visits to healthcare professionals in the last 30 days, median (IQR)	2 (0-3)	1.5 (0-3)	2 (1-4)	0.26
Number of visits by nurse & home help in the last 30 days, median (IQR)	0 (0-2)	0 (0-2)	0 (0-2)	0.26

Notes:

a. P value was based on chi-squared test for categorical variables; independent sample t-test for continuous variables; and 2-group Wilcoxon rank-sum tests. SD = Standard Deviation, IQR = Inter-Quartile Range.

b. Total number of hours spent care giving in the last 30 days is the sum of hours spent assisting activities of daily living (ADLs), activities of instrumental activities of daily living (IADL) and supervising the older person with mild cognitive impairment.

c. Number of workdays affected by care duty in the last 30 days was only compared amongst carers who worked for pay.

d. Total number of visits that use health resources in the last 30 days is the sum of visits to health professionals, by nurse and home help, and hospital and emergency department (ED) admissions.

Supplementary file. 'Taking Charge' after a diagnosis of cognitive impairment or dementia: A randomised controlled trial

Table S10. Project evaluation

Question	Responses	n (% of n)
Was the project clearly explained to you from the time we first contacted you?[a]	No	6 (4.1%)
	Yes	127 (86.4%)
	Unknown	14 (9.5%)
Overall, how much do you think you benefited from participation in this project?[a]	Not at all	13 (8.8%)
	Some	65 (44.2%)
	A great deal	42 (28.6%)
	Unknown	27 (18.4%)
How much did participation in this project help you feel more hopeful about your future health?[a]	Not at all	24 (16.3%)
	Some	64 (43.5%)
	A great deal	30 (20.4%)
	Unknown	29 (19.7%)
How much did participation in the project help enhance your ability to understand how to achieve a healthy lifestyle?[b]	Not at all	20 (13.7%)
	Some	76 (52.1%)
	A great deal	29 (19.9%)
	Unknown	21 (14.4%)
Did the project require too much work or effort? [b]	Not at all	128 (87.7%)
	Some	13 (8.9%)
	A great deal	0 (0.0%)
	Unknown	5 (3.4%)
Would you recommend this project to others in similar situations? [b]	No	8 (5.5%)
	Yes	115 (78.8%)
	Unknown	23 (15.8%)
**Additional comments about project:**<br>“Good to have someone take the time to listen and actually talk with.” (P9, Control)<br>“Program is a great help to anyone in this situation, helps [me] to think further and actually take action for [my] future.” (P27, Take Charge)<br>“Answered as "not at all" because I already knew/did a lot of the things taught but do think others who don't know these things would find it useful” (P14, Take Charge)<br>“Everything been good so far! Has made him consciously remember that there is always something good and positive to do. Considers himself a positive person but has made him even more positive about things at the moment.” (P49, Control)<br>“[Therapist] was lovely to talk with. Very professional and caring. Good communicator. Gave us some helpful ideas on living with dementia. Assisted in life skills. Happy to have her in our home again.” (P63, Control)<br>“Participant noted that he was quite frustrated with some of the questions in the program, especially the question asking him to draw his activities. This made him anxious as he does not think he has good drawing skills and did not understand why he couldn't just describe the activity instead. Would prefer the option of being able to talk through answers rather than draw them.” (P76, Take Charge)<br>“Considering the way things are going in terms of health and the future then this is definitely a better option and program to be in than not being part of it.” (P82, Control)<br>“I learned a lot about what to do and how to achieve healthier lifestyle, but actually doing those things etc is what's challenging and I feel like I need support in.” (P84, Take Charge)<br>“The project gave me insight into what my blessings are and allowed me to reflect. Before this I took good health for granted. The project has helped me to manage better and to come to terms with things. It made me think” (P136, Control)		
Notes: a, n=147 responses; b, n=146 responses.		

Supplementary file. 'Taking Charge' after a diagnosis of cognitive impairment or dementia: A randomised controlled trial

Interview guide

Background:

- Living arrangements
- Age
- Diagnosis
- Time since last saw Take Charge therapist

You recently participated in the Take Charge program. The program consisted of a health professional [name] visiting you twice about 6 weeks apart and going through a booklet with you. You would have talked a bit about your values and what was important to you. You might have set some goals, maybe talked about getting into some activities that you had stopped doing, and talked about how you could make the most of your life. Does that sound familiar at all?

1. Let's talk about expectations first, when you heard about the program.
    a. Why did you decide to take part?
    b. What did you hope to gain from the program?

2. What about experiences with the program?
    a. The health professional [name] might have worked through a booklet with you. How did you find completing that?
    b. You might have also set some goals for the future? What was that like for you? Have you set goals like that before?
    c. The health professional [name] might have also talked to you about things you used to do but haven't done in a while.. was there anything that you realised you would like to do again that you could maybe get back into doing?
    d. Were there other things that really stood out to you? For example some of the questions that she asked you?
    e. Were there specific things the therapist could help you with?
        i. *The most helpful aspects of the program? (Eg. timely, collaborative, practical, specific, easy to understand, relevant)*
        ii. *The least helpful aspects (Eg. too prescriptive (i.e. not tailored enough), too difficult, too invasive)*
    f. Did you have a chance to provide input into the goals that you set did you feel this was something that came more from the health professional [name]?

3. It has been *[xx time]* since you last saw the health professional [*name*]. How are things going now?
    a. Do you recall the goals you set?
    b. Have you been working towards the goals you set?
        i. *If so, how? If not, why would that be?*
    c. Has participating in this program affected the way you think about the future or your diagnosis?
        1. *If so, how? If not, why would that be?*

Do you have any advice on how to improve the program?
What is your overall satisfaction with the program

4. 1 – Not valuable I 2 - Somewhat valuable I 3 - Valuable I 4 – Very valuable

Supplementary file. 'Taking Charge' after a diagnosis of cognitive impairment or dementia: A randomised controlled trial

CONSORT 2010 checklist of information to include when reporting a randomised trial*

Section/Topic	Item No	Checklist item	Reported on page No
**Title and abstract**			
	1a	Identification as a randomised trial in the title	Page 1
	1b	Structured summary of trial design, methods, results, and conclusions (for specific guidance see CONSORT for abstracts)	Page 1
**Introduction**			
Background and objectives	2a	Scientific background and explanation of rationale	Page 2-3
	2b	Specific objectives or hypotheses	Page 3
**Methods**			
Trial design	3a	Description of trial design (such as parallel, factorial) including allocation ratio	Page 3
	3b	Important changes to methods after trial commencement (such as eligibility criteria), with reasons	N/A
Participants	4a	Eligibility criteria for participants	Page 3
	4b	Settings and locations where the data were collected	Page 3
Interventions	5	The interventions for each group with sufficient details to allow replication, including how and when they were actually administered	Page 4
Outcomes	6a	Completely defined pre-specified primary and secondary outcome measures, including how and when they were assessed	Page 5-6
	6b	Any changes to trial outcomes after the trial commenced, with reasons	N/A
Sample size	7a	How sample size was determined	Page 3
	7b	When applicable, explanation of any interim analyses and stopping guidelines	N/A
Randomisation:			
Sequence generation	8a	Method used to generate the random allocation sequence	Page 4
	8b	Type of randomisation; details of any restriction (such as blocking and block size)	Page 4
Allocation concealment mechanism	9	Mechanism used to implement the random allocation sequence (such as sequentially numbered containers), describing any steps taken to conceal the sequence until interventions were assigned	Page 4
Implementation	10	Who generated the random allocation sequence, who enrolled participants, and who assigned participants to interventions	Page 4-5

Blinding	11a	If done, who was blinded after assignment to interventions (for example, participants, care providers, those assessing outcomes) and how	Page 5
	11b	If relevant, description of the similarity of interventions	N/A
Statistical methods	12a	Statistical methods used to compare groups for primary and secondary outcomes	Page 5-6
	12b	Methods for additional analyses, such as subgroup analyses and adjusted analyses	Page 5-6
**Results**			
Participant flow (a diagram is strongly recommended)	13a	For each group, the numbers of participants who were randomly assigned, received intended treatment, and were analysed for the primary outcome	Page 6, Figure 1
	13b	For each group, losses and exclusions after randomisation, together with reasons	Page 6-7, Figure 1
Recruitment	14a	Dates defining the periods of recruitment and follow-up	Page 6
	14b	Why the trial ended or was stopped	N/A
Baseline data	15	A table showing baseline demographic and clinical characteristics for each group	Table 1
Numbers analysed	16	For each group, number of participants (denominator) included in each analysis and whether the analysis was by original assigned groups	Table 1 and Table S2
Outcomes and estimation	17a	For each primary and secondary outcome, results for each group, and the estimated effect size and its precision (such as 95% confidence interval)	Page 6-8, Tables 2-3, Supplementary Tables
	17b	For binary outcomes, presentation of both absolute and relative effect sizes is recommended	N/A
Ancillary analyses	18	Results of any other analyses performed, including subgroup analyses and adjusted analyses, distinguishing pre-specified from exploratory	Page 8, Supplementary Tables
Harms	19	All important harms or unintended effects in each group (for specific guidance see CONSORT for harms)	Page 7
**Discussion**			
Limitations	20	Trial limitations, addressing sources of potential bias, imprecision, and, if relevant, multiplicity of analyses	Page 9-11
Generalisability	21	Generalisability (external validity, applicability) of the trial findings	Page 9-11
Interpretation	22	Interpretation consistent with results, balancing benefits and harms, and considering other relevant evidence	Page 9-11
**Other information**			
Registration	23	Registration number and name of trial registry	Page 3

Protocol	24	Where the full trial protocol can be accessed, if available	Page 3
Funding	25	Sources of funding and other support (such as supply of drugs), role of funders	Page 12

Citation: Schulz KF, Altman DG, Moher D, for the CONSORT Group. CONSORT 2010 Statement: updated guidelines for reporting parallel group randomised trials. BMC Medicine. 2010;8:18.
© 2010 Schulz et al. This is an Open Access article distributed under the terms of the Creative Commons Attribution License (http://creativecommons.org/licenses/by/2.0), which permits unrestricted use, distribution, and reproduction in any medium, provided the original work is properly cited.

*We strongly recommend reading this statement in conjunction with the CONSORT 2010 Explanation and Elaboration for important clarifications on all the items. If relevant, we also recommend reading CONSORT extensions for cluster randomised trials, non-inferiority and equivalence trials, non-pharmacological treatments, herbal interventions, and pragmatic trials. Additional extensions are forthcoming: for those and for up-to-date references relevant to this checklist, see www.consort-statement.org.
